# Salivary Galectin-3 Levels Among Healthy Individuals, Patients With Periodontitis, Oral Potentially Malignant Disorders, and Oral Cancer: A Cross-Sectional Study

**DOI:** 10.7759/cureus.110717

**Published:** 2026-06-12

**Authors:** Saumya Singh, Girish Suragimath, Siddhartha Varma, Sameer A Zope, Vaishali N Mashalkar, Apurva V Kale, Anand Gudur

**Affiliations:** 1 Department of Periodontology, School of Dental Sciences, Krishna Vishwa Vidyapeeth (Deemed to be University), Karad, IND; 2 Department of Oncology, School, of Dental Sciences, Krishna Vishwa Vidyapeeth (Deemed to be University), Karad, IND

**Keywords:** galectin-3, oral potentially malignant disorders, oral squamous cell carcinoma, periodontitis, saliva biomarker

## Abstract

Background

Chronic inflammation and oral microbial imbalances contribute significantly to the development of oral potentially malignant disorders (OPMDs) and oral cancer. Galectin-3 (Gal-3), an inflammatory biomarker, is involved in immune response, cell death, angiogenesis, and cancer progression, making it a potential biomarker for both inflammatory and malignant conditions. Saliva enables non-invasive, convenient biomarker analysis for detecting inflammatory conditions.

Aim

To assess and compare salivary Gal-3 levels among healthy individuals, patients with periodontitis, OPMDs, and oral cancer, and to explore its potential association with inflammatory and malignant oral conditions.

Materials and methods

This cross-sectional study included 120 subjects of both genders, divided into four groups (n = 30 each): healthy controls; individuals with periodontitis; individuals with periodontitis and OPMDs; and those with periodontitis and oral cancer. Clinical periodontal parameters were documented, and unstimulated saliva samples were collected in a standardised manner. Salivary Gal-3 levels were measured using an Enzyme-Linked Immunosorbent Assay (ELISA). Appropriate statistical tests were performed to examine differences in Gal-3 levels between groups, and a p-value < 0.05 was considered significant.

Results

The mean age of the study population was 44.60, with significant differences among the groups. Most participants across all groups were males, but this was not statistically significant. There were statistically significant differences in periodontal parameters, i.e., probing pocket depth and clinical attachment loss, among the groups. Significant differences were observed in salivary Gal-3 levels among all groups, with the lowest in the healthy group and the highest in the periodontitis with oral cancer group. One-way analysis of variance (ANOVA) demonstrated statistically significant intergroup differences in salivary Gal-3 levels, F (3, 116) = 9.11, p < 0.001. However, given the cross-sectional design and potential confounding factors, these findings should be interpreted with caution.

Conclusion

Elevated salivary Gal-3 levels observed in OPMDs and oral cancer patients suggest a potential association with tumour progression and oral carcinogenesis. Salivary Gal-3 has the potential to serve as a non-invasive, adjunctive biomarker for early detection and risk assessment, particularly in high-risk individuals such as those with chronic periodontitis, OPMDs, and oral cancers. Further longitudinal studies are required to validate its clinical utility.

## Introduction

Oral cancer and oral potentially malignant disorders (OPMDs) are major global health problems, particularly in Southeast Asia, where tobacco use, alcohol consumption, and betel quid chewing are recognised as major risk factors. Oral cancer is a significant malignancy affecting the oral cavity, often arising from the epithelial lining of the oral mucosa and characterised by aggressive local invasion, considerable morbidity, and mortality. Despite advances in diagnostic techniques, early diagnosis remains challenging, and histopathological examination continues to be the gold standard for confirmation [1].

Chronic inflammation plays an important role in the initiation and progression of oral cancer [2]. Various OPMDs, such as leukoplakia and oral submucous fibrosis (OSMF), possess a significant risk of malignant transformation. In addition to OPMDs, periodontal disease, a chronic inflammatory condition associated with pathogenic microbial biofilms, has been increasingly linked with oral carcinogenesis. Persistent periodontal inflammation promotes the release of cytokines, reactive oxygen species, and other inflammatory mediators, thereby creating a favourable microenvironment for tumour initiation, progression, and malignant transformation [3].

The role of Galectin-3 (Gal-3) in maintaining epithelial integrity, regulating immune cells, and defending against microbial invasion suggests its important involvement in the pathogenesis and host immune response of periodontal disease. The Galectin family includes 15 identified proteins and is divided into three categories: prototypes with a single carbohydrate-recognition domain (CRD), tandem-repeat types with two CRDs, and chimaeras. Gal-3 is unique as the sole chimaera-type member, with an extended N-terminal region and a single CRD [4].

Gal-3 levels have been reported to increase significantly in chronic inflammatory conditions such as periodontitis, as well as in periodontitis with OPMDs and oral squamous cell carcinoma (OSCC), reflecting its role in inflammation, immune modulation, angiogenesis, tumour invasion, and metastatic progression. Compared with several other salivary biomarkers, Gal-3 has shown potential clinical relevance because of its involvement in both inflammatory and malignant pathways, making it a promising non-invasive salivary biomarker for the early detection, monitoring, and differentiation of periodontal disease, OPMDs, and oral cancer.

Saliva is a promising diagnostic medium due to its non-invasive, cost-effective, and accessible nature. There is a need to assess and correlate salivary Gal-3 levels among healthy and diseased groups to assess its potential as a biomarker. This exploratory study evaluates salivary Gal-3 levels in healthy individuals, patients with periodontitis, patients with periodontitis and OPMDs, and patients with oral cancer to assess its potential as a diagnostic and prognostic biomarker for distinguishing inflammatory oral diseases from malignant conditions.

## Materials and methods

Study design

This cross-sectional study was conducted at the Department of Periodontology, School of Dental Sciences, and the Department of Microbiology, Krishna Vishwa Vidyapeeth, Karad. The study was conducted between November 2024 and September 2025. All participants were informed about the objective of the study and procedures involved.

Ethical considerations

The study was conducted in accordance with the ethical principles of the Declaration of Helsinki. Ethical approval was obtained from the Institutional Ethics Committee of Krishna Vishwa Vidyapeeth, Karad (IEC No: KVV/IEC/10/2024). Written informed consent was obtained from all participants prior to enrolment in the study.

Sample size estimation

Sample size estimation was performed using G*Power software (Heinrich Heine University Düsseldorf, Düsseldorf, Germany), assuming an effect size of 0.40, an alpha level of 0.05, and 95% power, yielding a sample size of 120 participants (30 per group). No formal pilot study was conducted prior to the main investigation; however, all clinical procedures were standardised in accordance with established periodontal examination protocols.

Selection criteria for study subjects

Adults aged 18 years and above, regardless of gender, were included. Information regarding tobacco and areca nut use was obtained from all participants. Thirty subjects with no tobacco or areca nut use were considered the healthy subjects (group A). Thirty patients with generalised periodontitis, confirmed clinically and radiographically, were considered group B. Thirty patients diagnosed with OPMDs, such as leukoplakia, erythroplakia, proliferative verrucous leukoplakia, lichen planus, OSMF, lupus erythematosus, epidermolysis bullosa, and dyskeratosis congenita, were considered as group C. Thirty patients histopathologically diagnosed with oral cancer before the initiation of treatment were considered as group D. Individuals with systemic or metabolic diseases (such as diabetes or autoimmune disorders) were excluded. Exclusion also applied to those subjects taking medications affecting the periodontium, pregnant or lactating women, and oral cancer patients who had received prior treatment. Detailed quantification of tobacco exposure, including duration, frequency, and pack-years, was not performed.

Examiner calibration

All periodontal examinations were performed by a single trained examiner to ensure consistency in clinical measurements. Prior to commencement of the study, the examiner underwent calibration exercises using standard periodontal examination protocols.

Clinical examination of participants

A comprehensive periodontal examination was performed for all participants. Clinical parameters assessed included probing pocket depth (PPD), simplified oral hygiene index (OHI-S), and clinical attachment level (CAL). PPD and CAL were measured in millimetres at six sites per tooth using a UNC-15 periodontal probe. PPD was measured from the gingival margin to the base of the pocket. At the same time, CAL was recorded as the distance from the cemento-enamel junction to the base of the gingival sulcus. Orthopantomograms (OPGs) were obtained to assess alveolar bone loss. Periodontitis patients were staged and graded according to the 2017 American Academy of Periodontology World Workshop Classification of Periodontal and Peri-implant Diseases and Conditions [5].

Saliva sample collection

Unstimulated whole saliva samples (2 mL) were collected between 10:00 AM and 12:00 PM using the passive drooling technique. Participants were instructed to avoid eating, drinking, or performing oral hygiene at least one hour before sample collection. Clinical examination was completed at least one hour before saliva collection to avoid blood contamination. Subjects were asked to tilt their heads slightly and expectorate saliva into sterile tubes for over approximately five minutes until the required volume was obtained.

Sample storage

Saliva samples were immediately centrifuged at 1000 rpm for 15 minutes at 2-8°C to remove cellular debris and particulates. The supernatant was transferred into Eppendorf tubes (Eppendorf SE, Hamburg, Germany) and stored at −80°C until analysis.

Analysis of biomarker

Salivary Gal-3 levels were measured using the Human GAL-3 sandwich Enzyme-Linked Immunosorbent Assay (ELISA) principle (Cat. No. ELK2790; ELK Biotechnology Co., Ltd., Wuhan, China) following the manufacturer’s instructions. The assay sensitivity was 0.062 ng/mL with a detection range of 0.16-10 ng/mL. The kit demonstrated high specificity for Human GAL-3, with no significant cross-reactivity or interference observed with related analogues.

Statistical analysis

**Figure 1:**
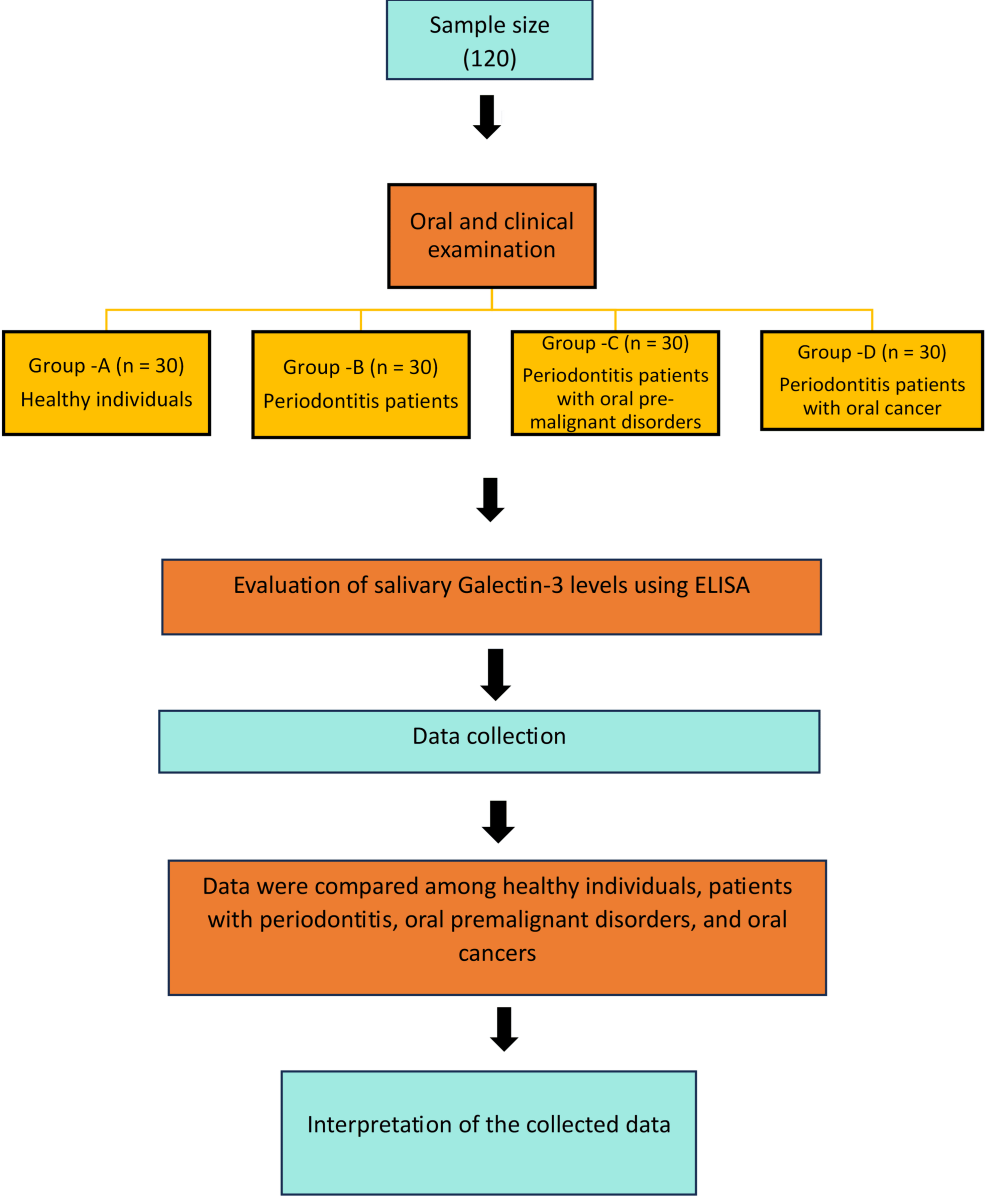
Summary of the study protocol.

Data were analysed using IBM SPSS Statistics for Windows, Version 25 (Released 2017; IBM Corp., Armonk, New York). Descriptive statistics were expressed as frequencies, percentages, means, standard deviations, standard errors, and 95% confidence intervals (CI). Normality of continuous variables was assessed using the Shapiro-Wilk test and visual inspection of Q-Q plots. Homogeneity of variance was evaluated using Levene’s test. Intergroup comparisons were performed using one-way analysis of variance (ANOVA), followed by Tukey’s post hoc test for pairwise comparisons. Effect size for ANOVA was reported using eta squared (η²). Pearson’s correlation coefficient was used to assess associations between periodontal clinical parameters and salivary Gal-3 levels after confirming linearity and normality assumptions. A p-value < 0.05 was considered statistically significant for all analyses (Figure 1).

## Results

The mean age of the study participants varied across the study groups, with Group A demonstrating the lowest mean age and Groups C and D showing comparatively higher mean ages. The mean age was 29.65 ± 6.82 years in Group A, 46.78 ± 8.94 years in Group B, 51.12 ± 7.65 years in Group C, and 50.84 ± 7.72 years in Group D. Intergroup comparison using one-way ANOVA revealed a statistically significant difference in mean age among the groups (F(3,116) = 21.376, p < 0.001), with healthy controls being younger than the disease groups. Males predominated across all study groups, with the highest proportion observed in Group C (86.67%). However, no statistically significant difference in gender distribution was observed among the groups (χ² = 5.412, p = 0.144) (Table 1).

**Table 1:** Comparison of demographic characteristics (age) among study groups using one-way ANOVA. df = degrees of freedom.

Group	Mean age ± SD (years)	SE	Range (years)	F value	df	η²	p-value
Group A	29.65 ± 6.82	1.25	20.40-38.90				
Group B	46.78 ± 8.94	1.63	34.20-58.60	21.376	(3,116)	0.356	<0.001*
Group C	51.12 ± 7.65	1.40	41.00-60.00				
Group D	50.84 ± 7.72	1.41	40.85-59.90				

Group A demonstrated the lowest mean values for all periodontal parameters. In contrast, Group C exhibited the highest mean values, followed by Groups B and D. The mean OHI-S scores were 0.85 ± 0.32 in Group A, 2.35 ± 0.52 in Group B, 3.15 ± 0.58 in Group C, and 2.05 ± 0.42 in Group D, showing a statistically highly significant difference among the groups (F(3,116) = 503.42, p < 0.001). Similarly, the mean Russell’s Index scores were 0.25 ± 0.12 in Group A, 1.95 ± 0.42 in Group B, 3.45 ± 0.62 in Group C, and 2.55 ± 0.48 in Group D, with statistically significant intergroup differences (F(3,116) = 569.81, p < 0.001). The mean PPD was 1.6 ± 0.42 mm in Group A, 3.1 ± 0.58 mm in Group B, 4.7 ± 0.72 mm in Group C, and 2.9 ± 0.52 mm in Group D, demonstrating statistically significant variation among the groups (F(3,116) = 472.64, p < 0.001). Likewise, the mean CAL was 0.6 ± 0.22 mm in Group A, 2.1 ± 0.48 mm in Group B, 3.6 ± 0.62 mm in Group C, and 2.4 ± 0.42 mm in Group D, with statistically highly significant differences observed between the groups (F(3,116) = 196.37, p < 0.001). Overall, the differences in periodontal clinical parameters demonstrated very large effect sizes (η² range: 0.84-0.94), indicating substantial intergroup variation (Table 2).

**Table 2:** Comparison of periodontal clinical parameters among study groups using one-way ANOVA. *p-value < 0.05: Level of statistical significance. OHI: oral hygiene index; PPD: probing pocket depth; CAL: clinical attachment level; CI: confidence interval; df: degrees of freedom.

Parameter	Group	Mean ± SD	Std. Error	95% CI Lower Bound	95% CI Upper Bound	Minimum	Maximum	ANOVA F-value	df	η²	p-Value
OHI-S	A	0.85 ± 0.32	0.058	0.73	0.97	0.3	1.6	503.42	(3,116)	0.93	<0.001*
	B	2.35 ± 0.52	0.098	2.15	2.55	1.4	3.6				
	C	3.15 ± 0.58	0.109	2.92	3.38	2.1	4.3				
	D	2.05 ± 0.42	0.078	1.89	2.21	1.2	2.8				
Russell’s Index	A	0.25 ± 0.12	0.022	0.20	0.30	0.0	0.5	569.81	(3,116)	0.94	<0.001*
	B	1.95 ± 0.42	0.078	1.79	2.11	1.2	2.8				
	C	3.45 ± 0.62	0.117	3.21	3.69	2.4	4.8				
	D	2.55 ± 0.48	0.090	2.37	2.73	1.8	3.6				
PPD (mm)	A	1.6 ± 0.42	0.077	1.44	1.76	1.0	2.3	472.64	(3,116)	0.92	<0.001*
	B	3.1 ± 0.58	0.106	2.88	3.32	2.1	4.3				
	C	4.7 ± 0.72	0.136	4.42	4.98	3.4	6.1				
	D	2.9 ± 0.52	0.097	2.70	3.10	2.0	3.8				
CAL (mm)	A	0.6 ± 0.22	0.040	0.52	0.68	0.2	1.1	196.37	(3,116)	0.84	<0.001*
	B	2.1 ± 0.48	0.090	1.92	2.28	1.1	3.1				
	C	3.6 ± 0.62	0.117	3.36	3.84	2.6	4.8				
	D	2.4 ± 0.42	0.078	2.24	2.56	1.7	3.4				

Post hoc intergroup comparisons of periodontal clinical parameters revealed statistically significant differences across study groups. For OHI-S, statistically significant differences were observed across all intergroup comparisons (p < 0.001). The mean differences ranged from −2.30 between Groups A and C to 1.10 between Groups C and D. One-way ANOVA demonstrated a statistically significant overall difference among the groups, F(3,116) = 503.42, p < 0.001. Similarly, Russell’s Periodontal Index demonstrated statistically significant differences across all intergroup comparisons, with mean differences ranging from −3.20 between Groups A and C to 0.90 between Groups C and D (p < 0.001). The overall difference among the groups was statistically significant, F(3,116) = 569.81, p < 0.001. PPD differed significantly across all study groups (p < 0.001). The mean differences ranged from −3.10 between Groups A and C to 1.80 between Groups C and D. One-way ANOVA demonstrated a statistically significant overall intergroup difference, F(3,116) = 472.64, p < 0.001. Likewise, CAL demonstrated statistically significant differences between all study groups. The mean differences ranged from −3.00 between Groups A and C to 1.20 between Groups C and D. The difference between Groups B and D was comparatively smaller but remained statistically significant (mean difference = −0.30, p < 0.05). The overall difference among groups was statistically significant, F(3,116) = 196.37, p < 0.001 (Table 3).

**Table 3:** Intergroup comparison between OHI, Russell’s index, PPD, and CAL using Tukey’s post hoc test for periodontal clinical parameters. *p-value < 0.05: Level of statistical significance. OHI: oral hygiene index; PPD: probing pocket depth; CAL: clinical attachment level; CI: confidence interval; df: degrees of freedom.

Parameter	Comparison	Mean difference (I-J)	SE	95% CI lower	95% CI upper	F value	df	p-value
OHI-S	A vs B	-1.50	0.043	-1.66	-1.34	503.42	(3,116)	<0.001*
	A vs C	-2.30	0.043	-2.46	-2.14			<0.001*
	A vs D	-1.20	0.043	-1.36	-1.04			<0.001*
	B vs C	-0.80	0.043	-0.96	-0.64			<0.001*
	B vs D	0.30	0.043	0.14	0.46			<0.001*
	C vs D	1.10	0.043	0.94	1.26			<0.001*
Russell’s Periodontal Index	A vs B	-1.70	0.039	-1.85	-1.56	569.81	(3,116)	<0.001*
	A vs C	-3.20	0.039	-3.35	-3.06			<0.001*
	A vs D	-2.30	0.039	-2.45	-2.16			<0.001*
	B vs C	-1.50	0.039	-1.65	-1.36			<0.001*
	B vs D	-0.60	0.039	-0.75	-0.46			<0.001*
	C vs D	0.90	0.039	0.75	1.04			<0.001*
PPD (mm)	A vs B	-1.50	0.049	-1.68	-1.32	472.64	(3,116)	<0.001*
	A vs C	-3.10	0.049	-3.28	-2.92			<0.001*
	A vs D	-1.30	0.049	-1.48	-1.12			<0.001*
	B vs C	-1.60	0.049	-1.78	-1.42			<0.001*
	B vs D	0.20	0.049	0.02	0.38			<0.001*
	C vs D	1.80	0.049	1.62	1.98			<0.001*
CAL (mm)	A vs B	-1.50	0.077	-1.78	-1.22	196.37	(3,116)	<0.001*
	A vs C	-3.00	0.077	-3.28	-2.72			<0.001*
	A vs D	-1.80	0.077	-2.08	-1.52			<0.001*
	B vs C	-1.50	0.077	-1.78	-1.22			<0.001*
	B vs D	-0.30	0.077	-0.58	-0.02			<0.05*
	C vs D	1.20	0.077	0.92	1.48			<0.001*

Pearson’s correlation analysis demonstrated strong statistically significant positive correlations between periodontal clinical parameters and salivary Gal-3 levels. OHI-S (r = 0.801, p < 0.001), Russell’s Index (r = 0.842, p < 0.001), PPD (r = 0.861, p < 0.001), and CAL (r = 0.826, p < 0.001) exhibited strong positive correlations with salivary Gal-3 levels, indicating that worsening periodontal status was associated with increased salivary Gal-3 concentrations (Table 4).

**Table 4:** Pearson correlation analysis between periodontal clinical parameters and salivary Gal-3 levels among the study participants. *p-value < 0.05: Level of statistical significance. CI: confidence interval; df: degrees of freedom; OHI-S: simplified oral hygiene index; PPD: probing pocket depth; CAL: clinical attachment level; Gal-3: Galectin-3.

Parameters	Pearson correlation (r)	95% CI	p-value
OHI-S vs Russell’s Index	0.957*	0.938-0.970	<0.001
OHI-S vs PPD	0.948*	0.924-0.964	<0.001
OHI-S vs CAL	0.904*	0.864-0.932	<0.001
Russell’s Index vs PPD	0.979*	0.969-0.986	<0.001
Russell’s Index vs CAL	0.944*	0.918-0.961	<0.001
PPD vs CAL	0.953*	0.932-0.967	<0.001
Gal-3 vs OHI-S	0.801*	0.723-0.857	<0.001
Gal-3 vs Russell’s Index	0.842*	0.775-0.889	<0.001
Gal-3 vs PPD	0.861*	0.800-0.902	<0.001
Gal-3 vs CAL	0.826*	0.754-0.878	<0.001

**Figure 2:**
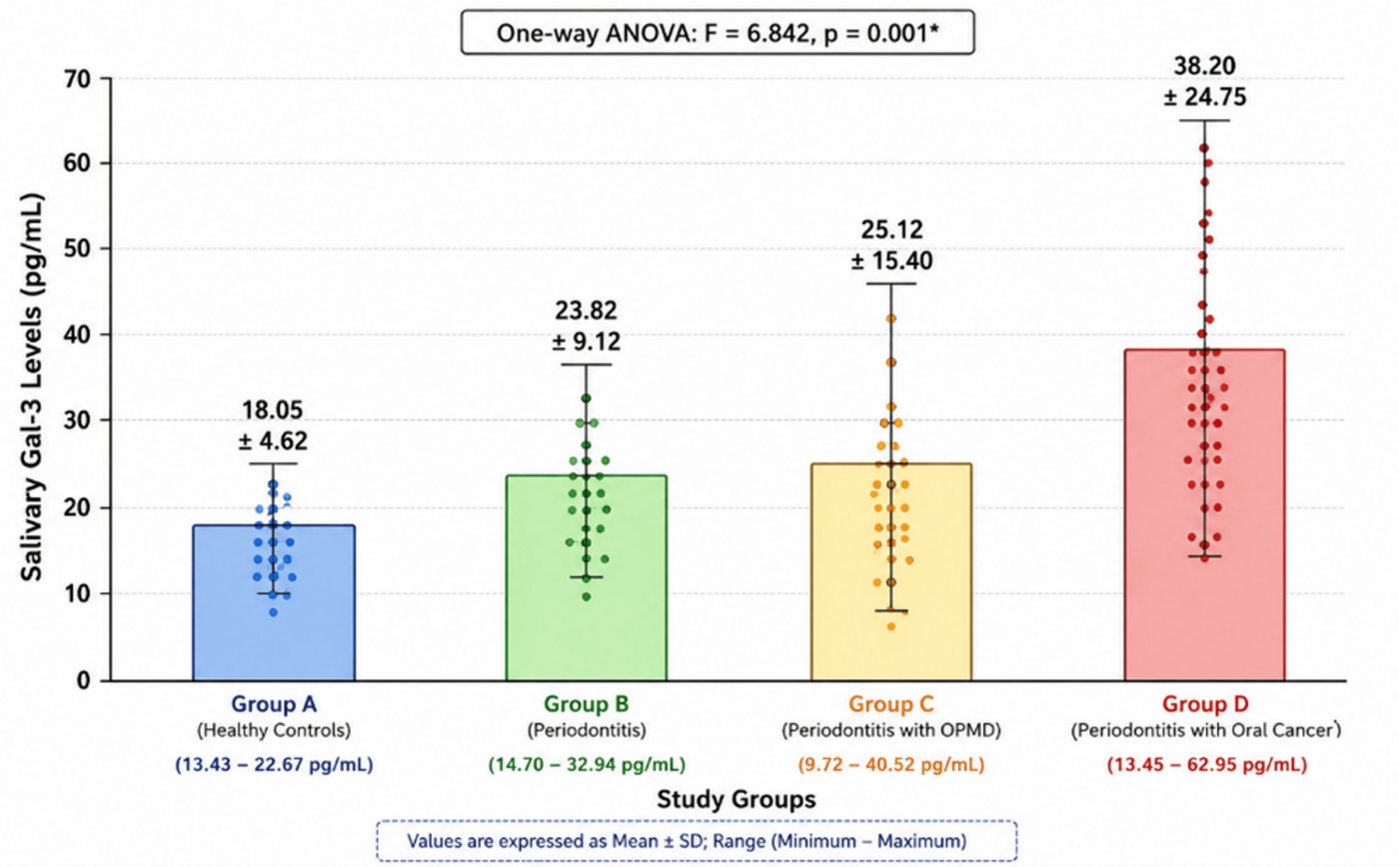
Comparison of mean salivary levels of Gal-3 among the groups. Gal-3: Galectin-3.

Salivary Gal-3 levels differed significantly among the study groups. Group A demonstrated the lowest mean (18.05 ± 4.62 pg/mL), with values ranging from 13.43 to 22.67 pg/mL. Group B showed a higher mean value of 23.82 ± 9.12 pg/mL (range: 14.70-32.94 pg/mL), while Group C demonstrated a mean value of 25.12 ± 15.40 pg/mL (range: 9.72-40.52 pg/mL). Group D exhibited the highest levels (38.20 ± 24.75 pg/mL), with values ranging from 13.45 to 62.95 pg/mL. After confirming the data distribution characteristics, a one-way ANOVA demonstrated statistically significant intergroup differences in salivary Gal-3 levels, F(3,116) = 9.11, p < 0.001, η² = 0.191, indicating a large effect size. Post hoc analysis revealed significantly lower salivary Gal-3 levels in Group A compared with Groups B, C, and D (p < 0.001). Group D demonstrated the highest salivary Gal-3 levels among all groups, whereas the difference between Groups B and C was comparatively smaller. These findings indicate elevated salivary Gal-3 levels in inflammatory, premalignant, and malignant oral conditions (Figure 2).

**Figure 3:**
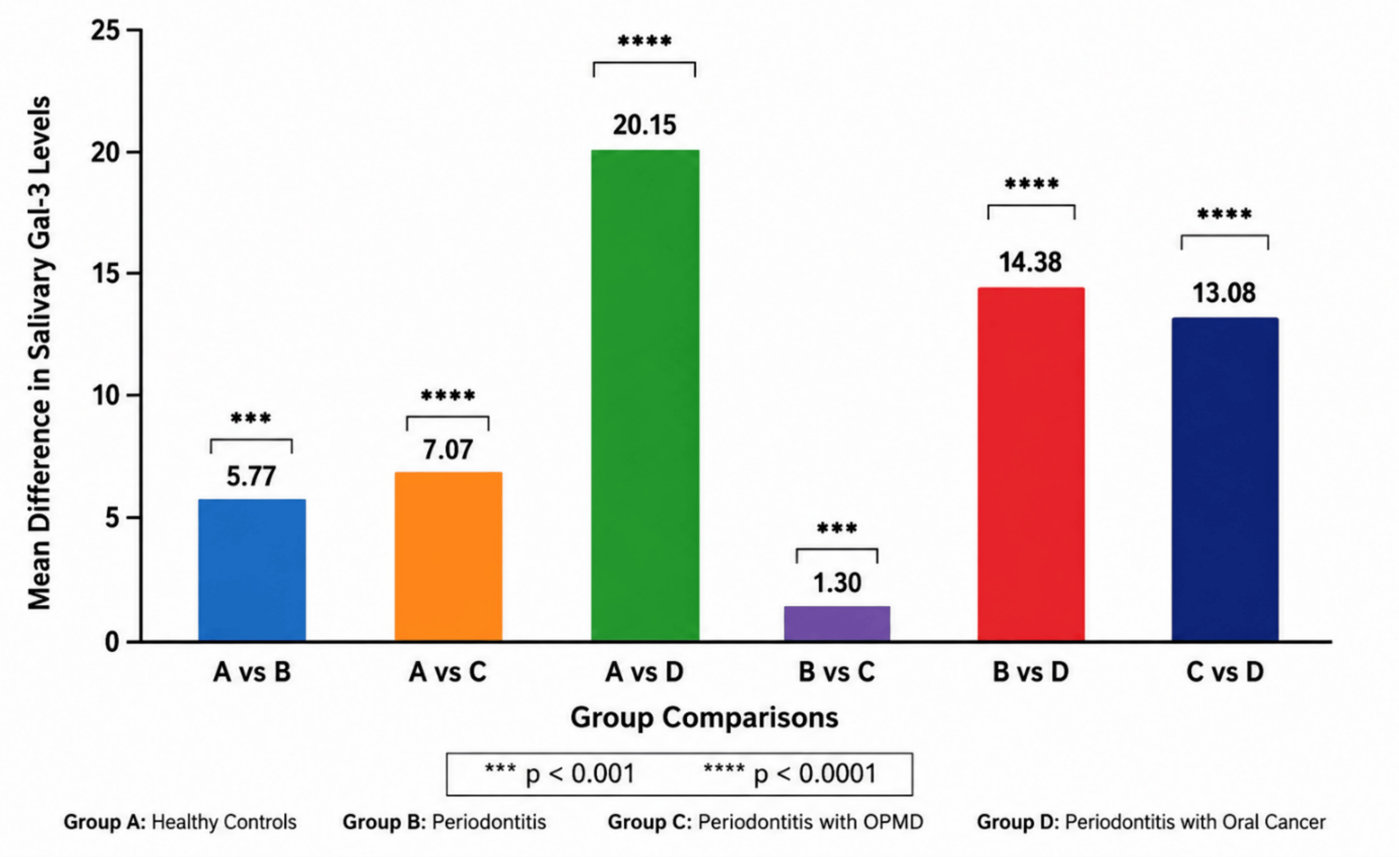
Post hoc analysis of salivary Gal-3 levels between various groups. Gal-3: Galectin-3.

Post hoc analysis revealed statistically significant differences in salivary Gal-3 levels among all study groups. Group A showed significant differences when compared with Group B (mean difference = 5.77, p < 0.001), Group C (7.07, p < 0.001), and Group D (20.15, p < 0.001). Significant differences were also observed between Group B and Group C (1.30, p < 0.001), Group B and Group D (14.38, p < 0.001), and Group C and Group D (13.08, p < 0.001). The greatest difference was observed between healthy controls (Group A) and oral cancer patients (Group D), while the smallest difference was observed between Groups B and C (Figure 3).

## Discussion

The present study evaluated salivary Gal-3 levels in healthy individuals, patients with periodontitis, patients with periodontitis and OPMD, and patients with periodontitis and oral carcinoma. Elevated salivary Gal-3 levels observed among diseased groups may reflect increased inflammatory and tumour-associated activity across the study groups, possibly due to enhanced Gal-3 production by inflammatory and tumour-associated cells during chronic inflammation and carcinogenesis [6,7]. Gal-3 is primarily produced by activated macrophages, neutrophils, epithelial cells, fibroblasts, and tumour cells, all of which contribute to immune regulation, cytokine release, tissue destruction, and tumour progression [8]. Persistent periodontal inflammation and microbial dysbiosis may stimulate increased Gal-3 expression, thereby promoting disease progression from periodontitis to periodontitis with OPMDs and oral carcinoma. Guo et al. and Liu and Rabinovich highlighted its role in tumour progression, invasion, and metastasis [9,10].

The mean age was higher in Groups B, C, and D than in Group A. Advancing age is associated with greater periodontal destruction, increased inflammatory burden, and a higher risk of OPMDs and oral cancer. Previous studies by Albandar et al. and Eke et al. demonstrated that cumulative exposure to microbial, environmental, and behavioural risk factors contributes to increased periodontal destruction with age [11,12]. Similarly, Warnakulasuriya and Petersen reported a greater prevalence of OPMDs and oral cancer among older individuals [13,14]. Therefore, age may have contributed to the observed elevation in salivary Gal-3 levels in diseased groups and should be considered a potential confounding factor when interpreting the findings. Future studies utilising age-matched populations and multivariable statistical adjustment are recommended to better isolate the independent effect of disease status on Gal-3 expression. Male predominance was observed in all groups, although the difference in gender predilection was not statistically significant. Previous studies by Clark et al. and Petti et al. reported a higher prevalence of periodontal disease and oral cancer among males, mainly due to tobacco use, alcohol consumption, and poor oral hygiene practices [15,16]. However, the present study suggests that behavioural and environmental factors may have a greater role in disease progression than gender alone.

Assessment of periodontal clinical parameters demonstrated significantly higher OHI-S, Russell’s Periodontal Index, PPD, and CAL values in diseased groups compared with healthy controls. Group C exhibited the highest periodontal destruction, indicating a severe inflammatory burden. These observations are consistent with the classical experimental gingivitis studies by Loe et al., who demonstrated that plaque is the primary etiological factor in gingival inflammation [17]. Greene and Vermillion further validated the OHI as a reliable indicator of oral hygiene status and periodontal disease severity [18]. The progressive increase in periodontal parameters observed in the present study reflects worsening periodontal status and chronic inflammation across the study groups. The present study demonstrated significantly elevated salivary Gal-3 levels in patients with periodontitis compared with healthy controls. Increased Gal-3 expression in periodontal disease may be associated with the activation of inflammatory pathways involving macrophages, neutrophils, cytokines, and matrix metalloproteinases. Sano et al. reported that Gal-3 functions as a chemoattractant for monocytes and macrophages, thereby amplifying inflammatory responses [19]. Similarly, Hendek et al. observed a reduction in Gal-3 levels following periodontal therapy, supporting its role as an inflammatory biomarker [20]. The elevated Gal-3 levels observed in patients with periodontitis in the present study therefore suggest an active host inflammatory response and periodontal tissue destruction.

Group C periodontitis patients with OPMD had higher salivary Gal-3 levels than healthy controls and periodontitis patients without OPMD. Chronic inflammation has been recognised as a critical factor in the malignant transformation of OPMD [21]. Persistent inflammatory stimuli promote cytokine release, oxidative stress, epithelial dysplasia, and genomic instability, thereby facilitating carcinogenesis. Guo et al. reported increased Gal-3 expression in inflammatory oral lesions and suggested a role for Gal-3 in modulating the tumour microenvironment and regulating the immune response [8]. Salivary Gal-3 concentrations were highest in individuals diagnosed with oral carcinoma, suggesting a strong association between Gal-3 and malignant transformation. Gal-3 has been reported to participate in several biological pathways associated with tumour growth and progression, as well as in processes such as preventing programmed cell death, promoting the formation of new blood vessels, aiding tissue invasion, and facilitating the spread of cancer cells to other parts of the body [9]. Liu and Rabinovich described Gal-3 as a multifunctional molecule involved in tumour progression and metastatic spread [10]. Weber et al. further demonstrated increased Gal-3 expression in aggressive OSCC lesions and its association with tumour invasiveness and poor prognosis [22]. The marked elevation of Gal-3 in Group D in the present study may therefore reflect advanced tumour activity and tumour-associated inflammatory responses.

The progressive increase in salivary Gal-3 levels from healthy individuals to patients with oral carcinoma observed in the present study highlights the close relationship between chronic inflammation and carcinogenesis. Persistent periodontal inflammation may create a tumour-promoting microenvironment by releasing inflammatory cytokines, reactive oxygen species, and microbial toxins, as well as by activating oncogenic pathways. Hajishengallis et al. explained that dysregulated host immune responses and chronic microbial dysbiosis contribute significantly to systemic inflammation and cancer progression [23]. These findings suggest a possible association between periodontitis and oral carcinogenesis. Certain periodontal bacteria, including *Porphyromonas gingivalis* and *Fusobacterium nucleatum*, may contribute to cancer development by evading immune defences, invading epithelial tissues, activating cytokines, and promoting tumour cell growth. Gallimidi et al. reported that periodontal pathogens accelerate tumour progression through chronic inflammatory signalling [24]. Lamont et al. demonstrated that *P. gingivalis* promotes oral cancer invasion by activating matrix metalloproteinases and dysregulating host immune pathways [25]. These mechanisms may partly explain the increase in Gal-3 levels observed in the diseased groups.

Saliva has gained increasing importance as a non-invasive diagnostic medium because it reflects both local and systemic pathological changes [26]. Salivary biomarker analysis offers advantages such as ease of collection, cost-effectiveness, patient compliance, and suitability for repeated monitoring [27]. Studies by Lee and Wong et al. demonstrated the usefulness of salivary biomarkers in the early detection of oral cancer and inflammatory diseases [28]. The present study further supports the utility of salivary Gal-3 as an adjunctive biomarker for differentiating inflammatory, premalignant, and malignant oral conditions.

Post hoc analysis in the present study demonstrated statistically significant differences in salivary Gal-3 levels across all study groups, with the largest difference observed between healthy individuals and patients with oral carcinoma. These findings indicate a gradual increase in inflammatory and tumour-associated burden from health to malignancy. Similar observations were reported by Tadbir et al. and Tokmak et al., who identified elevated Gal-3 expression in OSCC and suggested that it may serve as a prognostic marker [29,30]. However, some studies have reported variability in Gal-3 expression across tumour stage, lesion type, and methodological differences. Therefore, Gal-3 should preferably be interpreted in combination with clinical findings and other biomarkers. The OPMD and oral cancer groups included patients with different lesion types and clinicopathological characteristics. Variations in the biological behaviour, inflammatory activity, and malignant potential of individual lesions may influence Gal-3 expression. Because the number of participants within each lesion subtype was relatively small, lesion-specific subgroup analysis was not feasible. Future studies with larger sample sizes should evaluate Gal-3 levels across specific OPMD and oral cancer subtypes to determine whether distinct expression patterns exist among lesions.

Limitations and future perspectives

This study has certain limitations that should be considered while interpreting the findings. The cross-sectional design precludes determination of causal relationships and limits assessment of temporal changes in salivary Gal-3 levels. The study was conducted at a single centre with a relatively small sample size, which may limit the generalisability of the findings. A statistically significant age difference existed among the study groups, and age-related biological changes may have influenced periodontal status, inflammatory burden, and Gal-3 expression. Furthermore, confounding factors such as tobacco use, lifestyle habits, and environmental exposures were not completely controlled or quantified. The heterogeneity of lesions within the OPMD and oral cancer groups also represents a potential source of variability. Additionally, the coexistence of periodontitis in Groups C and D may have contributed to elevated salivary Gal-3 levels. Despite these limitations, the study has several strengths, including the use of a standardised saliva collection protocol, objective ELISA-based biomarker assessment, comprehensive periodontal evaluation, inclusion of multiple clinically relevant disease groups, and histopathological confirmation of oral cancer cases. Future multicentre longitudinal studies involving larger and age-matched populations are required to validate the diagnostic and prognostic utility of salivary Gal-3. Detailed assessment of tobacco exposure and lesion-specific subgroup analyses should also be incorporated. Evaluation of multiple salivary biomarkers alongside clinical and histopathological parameters may further improve the early detection, monitoring, and differentiation of inflammatory, premalignant, and malignant oral conditions.

## Conclusions

Within the limitations of this cross-sectional study, patients with periodontitis, periodontitis associated with OPMDs, and oral cancer demonstrated significantly higher salivary Gal-3 levels than healthy individuals. A progressive increase in Gal-3 levels was observed from inflammatory conditions to premalignant and malignant lesions, suggesting a possible association between Gal-3, chronic inflammation, tissue destruction, and oral carcinogenesis. However, the findings should be interpreted as associative rather than causal due to the study design and potential confounding factors, including age and tobacco exposure. Salivary Gal-3 may serve as a promising non-invasive adjunctive biomarker for identifying individuals at increased risk of oral disease progression. Further multicentre longitudinal studies are required before its routine clinical application can be recommended.
